# Design and Simulation Test of an Open D-Dot Voltage Sensor

**DOI:** 10.3390/s150923640

**Published:** 2015-09-17

**Authors:** Yunjie Bai, Jingang Wang, Gang Wei, Yongming Yang

**Affiliations:** 1 State Key Laboratory of Power Transmission Equipment & System Security and New Technology, Chongqing University, Chongqing 400044, China; E-Mails: byunjie@163.com (Y.B.); yangyym@cqu.edu.cn (Y.Y.); 2 Maintenance Branch of State Grid Chongqing Electric Power Company, Chongqing 400039, China; E-Mail: cqweig@163.com

**Keywords:** noncontact, Maxwell, open-ended, D-dot voltage sensor

## Abstract

Nowadays, sensor development focuses on miniaturization and non-contact measurement. According to the D-dot principle, a D-dot voltage sensor with a new structure was designed based on the differential D-dot sensor with a symmetrical structure, called an asymmetric open D-dot voltage sensor. It is easier to install. The electric field distribution of the sensor was analyzed through Ansoft Maxwell and an open D-dot voltage sensor was designed. This open D-voltage sensor is characteristic of accessible insulating strength and small electric field distortion. The steady and transient performance test under 10 kV-voltage reported satisfying performances of the designed open D-dot voltage sensor. It conforms to requirements for a smart grid measuring sensor in intelligence, miniaturization and facilitation.

## 1. Introduction

With the development of electrical power systems, higher requirements regarding accuracy, convenience and rapidity of power transformer are proposed. Following the principle of electric coupling, the D-dot voltage sensor’s characteristics can be described as: simple structure, small size, and no need of energy transfer [1,2,3,4]. It has attracted wide attention. However, the transfer function of traditional D-dot sensor differs under different frequencies. Therefore, based on the traditional D-dot sensor, a differential input is adopted, the ground terminal is removed, and a multi-electrodes parallel structure is employed to increase the mutual capacitance. In this way, the D-dot sensor could maintain the self-integral pattern under small frequency [5,6,7,8].

The self-integral D-dot voltage sensor has a simpler structure, a smaller size and a higher measuring bandwidth [5,6,7,8]. However, it is inconvenient in practical installation due to the symmetric structure. Therefore, structure of the differential D-dot voltage sensor was improved from the original symmetric one to an open sector structure.

### 1.1. Principle of the Differential D-Dot Sensor

**Figure 1:**
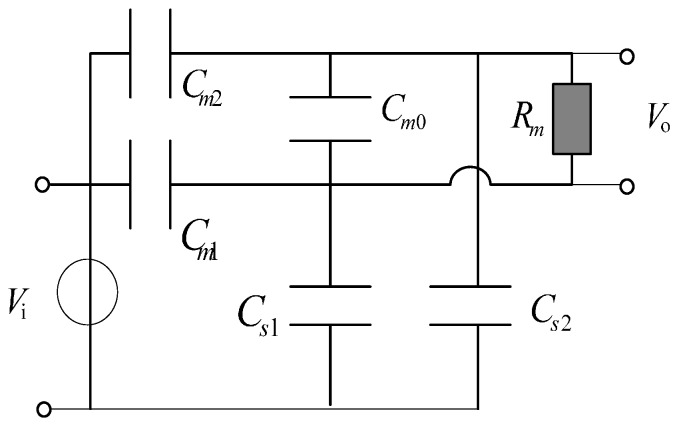
Equivalent circuit of the differential D-dot sensor.

The traditional D-dot voltage sensor will adopt different operating modes under different frequencies [9,10,11,12,13]. To maintain the self-integral mode, a differential D-dot sensor is designed [5,6,7,8]. Its equivalent circuits are shown in Figure 1.

In Figure 1, *C_m_*_2_ and *C_m_*_1_ are mutual capacitance between the measured conductor and the annular electrode. *C_s_*_1_ and *C_s_*_2_ are the stray capacitance from two annular electrodes to the ground. *C_m_*_0_ is the sum of the mutual capacitance between ring electrodes. *R_m_* is the equivalent input resistance that is used to measure the differential amplifier.

Its transfer function, magnitude-frequency characteristic and phase-frequency characteristic are:

(1) $$H(s)=\frac{V_{o}}{V_{i}}=\frac{sR_{m}C_{1}}{sR_{m}C_{2}+1}$$

(2) $$\left|H(\omega )\right|=\frac{R_{m}C_{1}}{\sqrt{{\left(R_{m}C_{2}\right)}^{2}+\frac{1}{\omega^{2}}}}$$

(3) $$∠H(\omega )=\frac{1}{\arctan R_{m}C_{2}\omega }$$

where

$$C_{1}=\frac{C_{m1}C_{s2}-C_{m2}C_{s1}}{C_{m1}+C_{m2}+C_{s1}+C_{s2}}$$

$$C_{2}=\frac{1}{\frac{1}{C_{m1}+C_{s1}}+\frac{1}{C_{m2}+C_{s2}}}+C_{m0}$$

More details about derivation of the transfer function are introduced in Reference [5]. *R_m_* of the differential amplifier is generally large, reaching tens or thousands of GΩ level. As shown in Equations (2) and (3), *R_m_C*_2_ will be much larger than the corner frequency (1/*ω*) when *C_m_*_0_ is increased through parallel structures, so that the sensor will keep working under the self-integral mode. It is known from Reference [5] that the bandwidth of the D-dot voltage sensor is very wide.

### 1.2. Symmetrical Differential D-Dot Sensor

**Figure 2:**
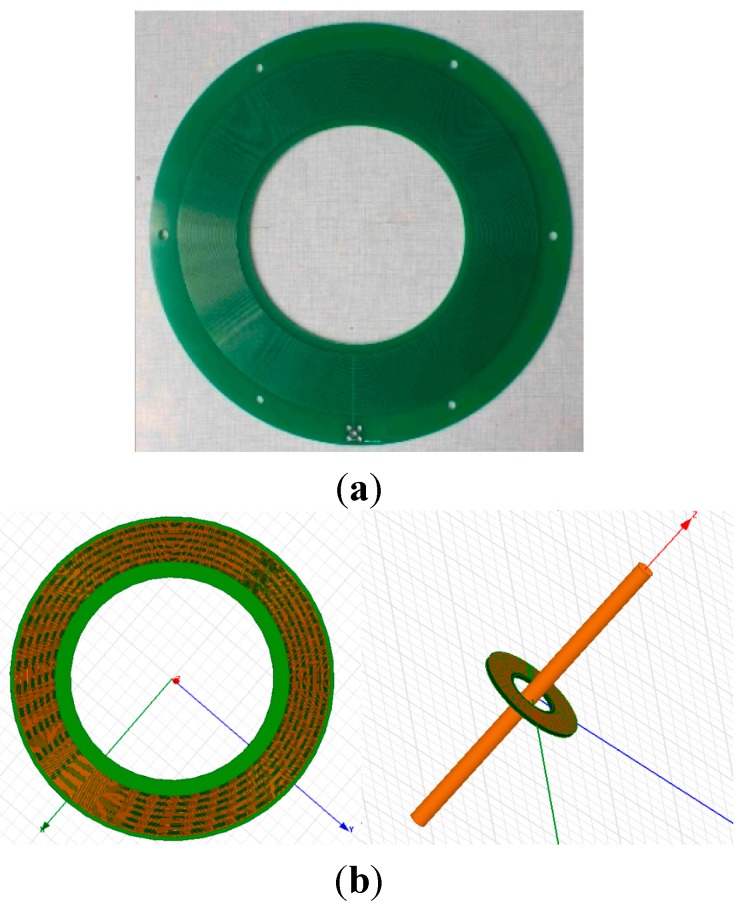
(**a**) Picture of a symmetrical differential D-dot sensor; (**b**) simulation model.

Figure 2 shows the real symmetrical differential D-dot sensor and its Ansoft simulation model.

This sensor has a simple structure, accessible insulating strength and good steady-state and transient-state performances. With a symmetric structure, it influences the electric field surrounding wires slightly.

However, it is difficult to make the measured wire run through the closed structure of this symmetrical differential D-dot sensor. When the symmetrical sensor is installed or removed, the measured wire also has to be moved. Therefore, based on the engineering practice, an open D-dot voltage sensor is designed. Compared to the symmetrical sensor, this open D-dot voltage sensor is superior in installation and removal. It is unnecessary to move the measured wire after the sensor is installed or removed.

### 1.3. Design of the Open D-Dot Voltage Mutual Transformer

#### Sensor Structure Design

**Figure 3:**
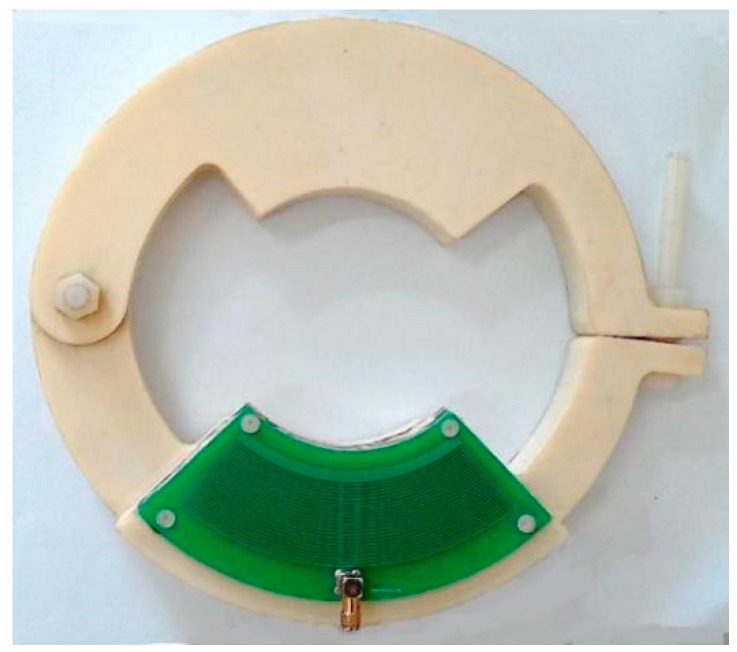
Open D-dot sensor.

A real open D-dot sensor is shown in Figure 3. It is mainly composed of an electrode on PCB and the epoxy insulation support which is served as the fixed end. The insulation support contains two parts which are fixed together by screws and could move freely, making the sensor easy to be installed. It could adjust surrounding electric fields and concentrate the strong field within the epoxy support. This reduces influences on the exterior electric field and the electric field surrounding the transmission lines [14]. Electrode and PCB panel in sector structure are fixed on the insulation support. 

As shown in Figure 1, the key of the differential D-dot sensor lies in *C_m_*_0_, the sum of the mutual capacitance between ring electrodes. Although electrodes are circular in symmetrical differential D-dot sensor and arcuate in an open D-dot sensor, there are mutual capacitances of electrodes [8]. So the mutual capacitance of an open D-dot sensor, which is the most important part, is consistent and similar with that of the symmetrical sensor. At the same time, the measure on the symmetrical sensor can also be used in open D-dot sensor. For example, multiple capacitors are connected in parallel to increase the total mutual capacitance [8].

In addition to the ring electrode, an insulating stand is also required, which is used to fix the ring electrode around the measured conductor. Given the good dielectric properties and high hardness of epoxy, epoxy resin could be used to produce this insulation bracket.

## 2. Simulation

### Electric Field Distortion and Insulating Strength

**Figure 4:**
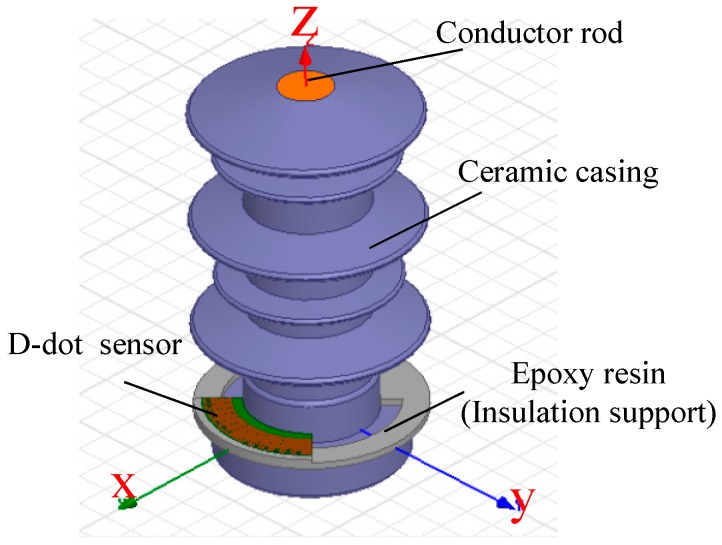
Finite element analysis model.

Due to the symmetric structure, the electric field of the primary differential sensor influences the electric field surrounding transmission lines slightly. However, the open D-dot sensor is asymmetrical. To prove that the open D-dot sensor could solve the electric distortion and insulation problems, a simulation calculation was conducted using Ansoft Maxwell, an electromagnetic field finite element computation software. The simulation model is shown as Figure 4.

In Figure 4, the gray part is the insulation support which is made of epoxy. The green part is PCB panel and the red part on the PCB panel is electrode. The open D-Dot sensor is installed outside the high-handed porcelain casing, responsible for measuring the voltage of the charged guide rod inside the casing. The porcelain casing and the conductor rod inside are not a part of the sensor design. Although they are not necessary for the sensor, they make the sensor easier to be installed and used. X-orientation is the symmetry axis of the sensor, namely, the orientation of electrode, while Y-orientation does not have electrode and is perpendicular to the X-orientation.

**Figure 5:**
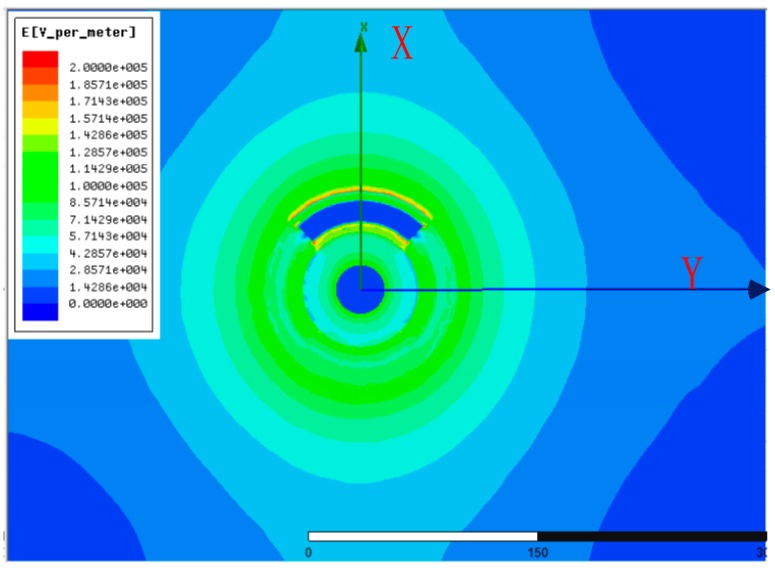
Electric field distribution surrounding the conductor.

Under 10 kV power-frequency voltage, electric field distribution of sensor and its surrounding areas are shown in Figure 5.

**Figure 6:**
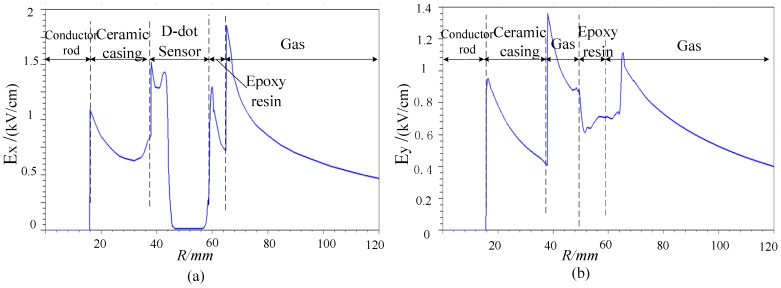
Distribution of electric field strength (**a**) X-orientation; (**b**) Y-orientation.

The magnitude of the total electric field vector along the x- and y- axis (orientation with and without electrode, shown in Figure 4 and Figure 5) from the origin are shown in Figure 6a,b, represented as *E_x_* and *E_y_*.

**Figure 7:**
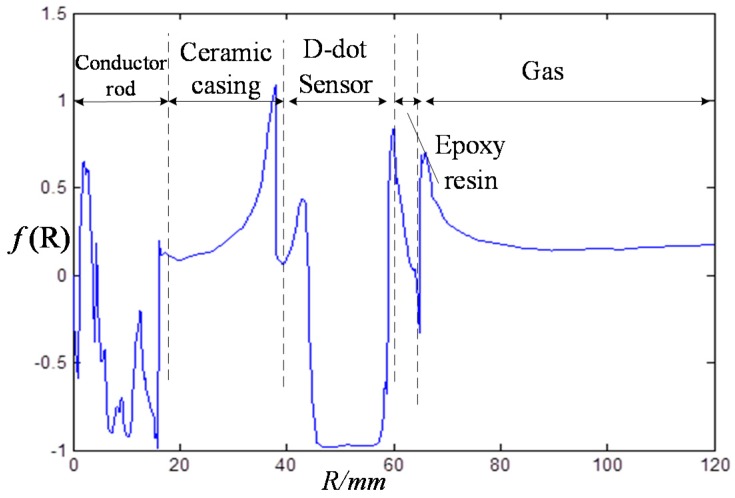
Gradient of electric field strength.

Places without sensor are far away from the electrode of sensor and electric field in these places will not suffer great influence [15]. Therefore, we assume that the electric field strength at places without sensor remain stable. To analyze the effect of sensor on electric field strength of wires, we managed to calculate the gradient of electric field strength of x-orientation to that of y-orientation (Figure 7).

The gradient of electric field strength of x-orientation to that of y-orientation is:

(4) $$f\left(\text{R}\right)=\frac{E_{x}-E_{y}}{E_{y}}$$

Indeed, Equation (4) has no physical meaning. However, it can be used to compare electric fields in two cases (with and without sensor) because in the absence of a sensor, the conductor and ceramic casing is axially symmetric.

(1) As shown in Figure 5 and Figure 7, sensor mainly influences the interior electrode and margin part of electrode. To the interior guide rod, the calculation of “*f*(R)” inside the conductor rod doesn’t have any meaning in physics, because the electric field strength inside the conductor rod is nearly zero. The interior and margin of the electrode influence other parts slightly due to the large gradient but small space. In a word, the sensor influences electric fields of the whole conductor slightly.

(2) As shown in Figure 6, the biggest electric field strength in the calculation area locates in the sensor-air interface, about 1.8 kV/cm. However, this is far smaller than the critical electric field strength of epoxy (200–300 kV/cm), ceramics (100–200 kV/cm) and air (25–30 kV/cm) [13]. To sum up, the asymmetrical open D-dot voltage sensor could offer satisfying insulating strength with certain margin.

## 3. Performance Test and Data Analysis of Sensor

### 3.1. Testing Platform 

**Figure 8:**
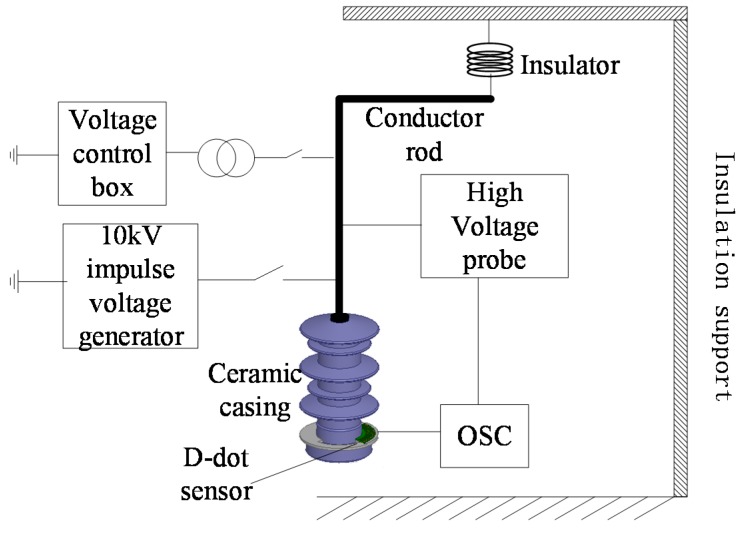
Experimental platform.

A platform (Figure 8) was constructed to test steady-state and transient-state performances of the designed sensor.

In this experimental platform, the guide rod is held in air by the insulation support and insulator [14]. The suspended guide rod passes through the transformer casing, while the sensor gets stuck outside the transformer casing. The transformer casing serves as an insulation protector and a sensor fixer. The stimulation on the guide rod is provided by a voltage control box through a step-up transformer, and standard lightning impulse voltage (1.2/50 μs, 10 kV peak value) is provided by a 10 kV impulse voltage generator. Voltage of the guide rod is measured directly by a high-voltage probe, which is used as the standard comparison signal. Its attenuation ratio is 1000:1. Then, signals measured by both high-voltage probe and sensor are input into the oscilloscope for comparison [16]. The signal from the sensor remains strong, so that it is unnecessary to undergo signal processing, such as amplification, filtering, *etc*.

### 3.2. Steady-State Error Test

#### 3.2.1. Power Frequency Steady-State Experiment

**Figure 9:**
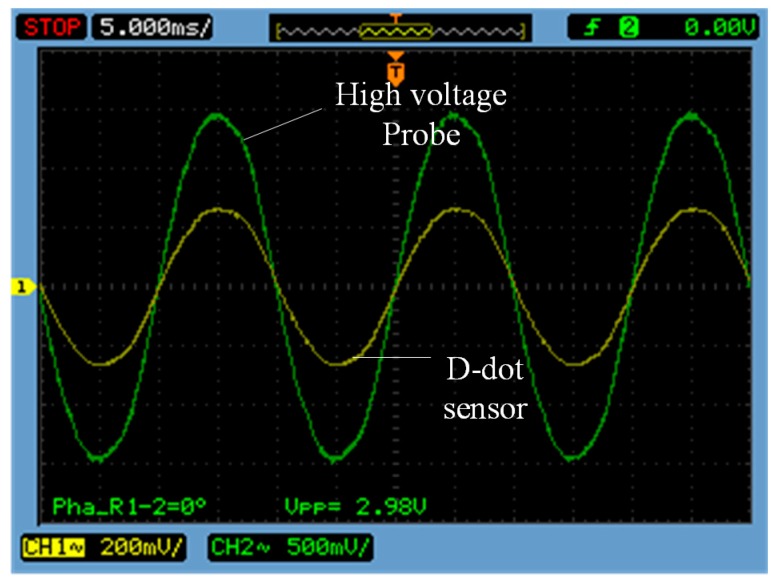
Waveform comparison chart in the 30% of rated voltage.

In the power frequency case, output voltages of the high-voltage probe and the sensor were measured when the effective voltage is equal to 10%, 30%, 50%, 80%, 100% and 120% of the rated voltage (10 kV) [17]. The waveform comparison chart of the high-voltage probe and the sensor at 30% of the rated voltage is shown in Figure 9.

Voltages measured by the high-voltage probe and the sensor at different voltages are shown in Table 1.

**Table 1:** Accuracy test results of D-dot sensor.

Measurement Points	*U_HV_*/kV	*U_D-_*_dot_/V	Ratio Error/%
10%Un	1.016	0.185	0.41
30%Un	2.98	0.540	−0.09
50%Un	5.002	0.903	−0.46
80%Un	8.032	1.45	−0.46
100%Un	9.995	1.815	0.13
120%Un	12.018	2.179	−0.03

*U_HV_* refers to the voltage measured by the high-voltage probe (converted into one power) and *U_D_*_-dot_ refers to the voltage measured by the mutual sensor. The ratio error is defined as [17]:

(5) $$\varepsilon \text{\%=}\frac{K_{n}U_{D\text{-dot}}-U_{HV}}{U_{HV}}\times 100\%$$

where *K_n_* refers to the voltage division ratio after the sensor is modified.

**Figure 10:**
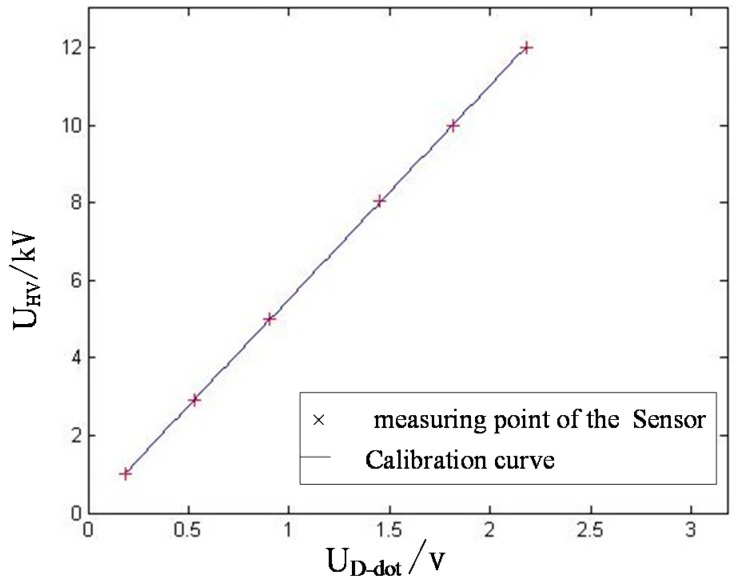
Output voltage calibration curve of mutual sensor and high-voltage probe at different voltages.

Data fitting was implemented (Figure 10). The fitting slope is the correction factor used to modify the voltage division ratio error of the sensor, valuing 5514. The one-time fitting error is 0.0005.

(1) In Figure 9, waveform distortion measured by the open self-integral D-dot voltage sensor remains low.

(2) In Table 1 and Figure 10, the linearity of the open self-integral D-dot voltage sensor remains great. However, compared to symmetric self-integral D-dot voltage sensor, the open self-integral D-dot voltage sensor has smaller amplitude and sensitivity [8]. This may be caused by the small equivalent area of the asymmetric structure or smaller parallel structure.

#### 3.2.2. Steady Harmonic Wave Test

Much of the equipment in an electrical system may generate ultraharmonics. These ultraharmonics will deteriorate electric energy quality and bring remarkable damage to the electric system [18]. Real-time measurement and countermeasures are necessary. Therefore, it is of great significance to measure harmonics accurately.

Self-integral D-dot voltage sensor has a large measuring bandwidth range. It could measure low-frequency voltage accurately and has stable transfer function to high-frequency waveform.

**Figure 11:**
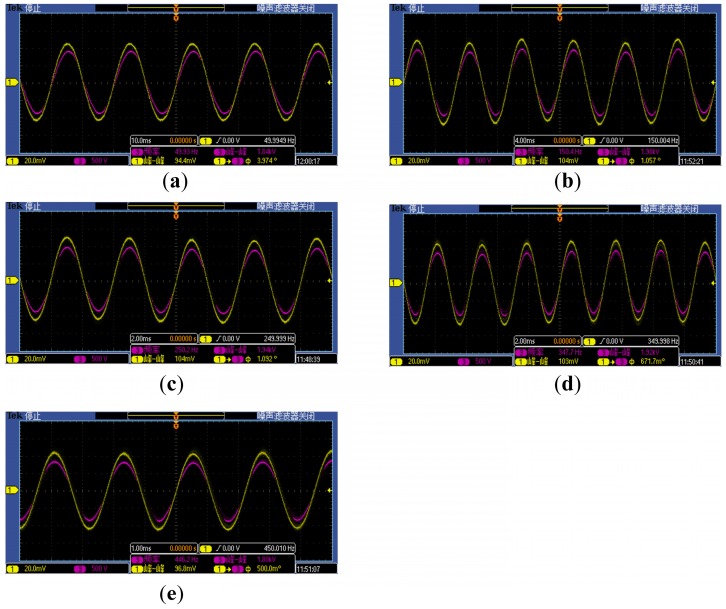
Waveform comparison chart at frequency (**a**) 50 Hz; (**b**) 150 Hz; (**c**) 250 Hz; (**d**) 350 Hz; (**e**) 450 Hz.

To test measuring accuracy of the open self-integral D-dot voltage sensor to harmonics, 50 Hz, 150 Hz, 250 Hz, 350 Hz and 450 Hz voltages were applied onto the guide rod, respectively, getting waveforms of the high-voltage probe and the sensor (Figure 11). The purple waveform is the high-voltage probe and the yellow one is the open self-integral D-dot voltage sensor.

The measuring amplitude and phase angle difference between the high-voltage probe and the sensor are shown in Table 2.

**Table 2:** Measuring accuracy of harmonic.

Measurement Points	*U_HV_*/kV	*U_D_*_-dot_/mV	Angular Difference (°)
50 Hz	1.84	94.4	3.974
150 Hz	1.98	104	1.057
250 Hz	1.94	104	1.092
350 Hz	1.92	103	0.6717
450 Hz	1.8	96.8	0.5

(1) In Figure 11, the waveform distortion of the open self-integral D-dot voltage sensor is small, ranging from 50 Hz to 450 Hz.

(2) In Chart 2, the angle between the waveform of the sensor and that of the high-voltage is small, basically remains about 1°. This indicates that the designed open self-integral D-dot voltage sensor has high measuring accuracy to fundamental wave and various waves, and could track various waveform component in electric energy system. However, compared to the symmetric self-integral D-dot voltage sensor [8], it reports bigger waveform phase angle difference. This may be caused by its smaller equivalent area of the asymmetric structure, or smaller parallel structure.

### 3.3. Transient-State Error Test

The structure of self-integral D-dot sensor is simple. It involves no sensor and no integration or differentiation in its transfer function, thus enabling to operate under the self-integral mode easily and respond to transient-state waveform quickly [1,2,3,4]. In this paper, a transient-state test of impulse voltage and switching operation was carried out. It confirmed that the open self-integral D-dot voltage sensor still maintains high measuring accuracy to transient-state waveform.

#### 3.3.1. Impulse Voltage Test

**Figure 12:**
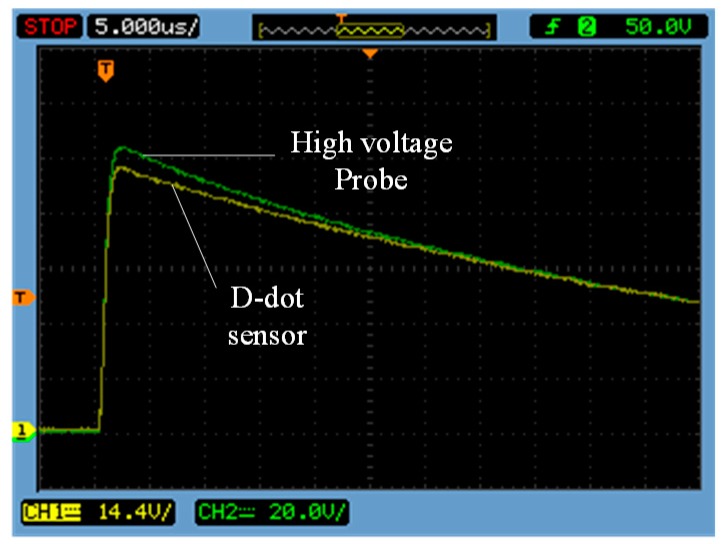
Measure waveform in 1.2/50 μs lightning wave.

A standard lightning impulse voltage (1.2/50 μs, 10 kV peak value) was generated by a 10 kV impulse voltage generator and then was applied onto the guide rod. Waveforms measured by the high-voltage probe and the sensor are shown in Figure 12. Green is the waveform measured by the high-voltage probe and yellow is the waveform measured by sensor.

In Figure 12, sensor is able to respond quickly to 1.2/50 μs standard lightning impulse voltage, without the occurrence of concussion. However, it still maintains good transient-state characteristics.

#### 3.3.2. Transient-State Test of Switching Operation

**Figure 13:**
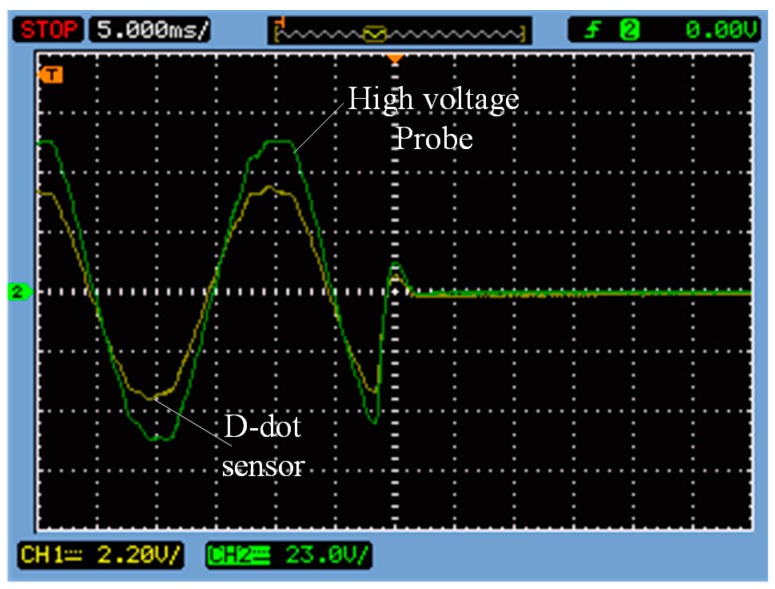
Transient waveform in the switching operation.

Add stable alternating voltage to the guide rod through voltage control box and step-up transformer, then switch off the added voltage suddenly. The waveform from stable phase to off voltage is shown in Figure 13. It can be seen that the waveform measured by the sensor decreases immediately [19], without the occurrence of concussion. However, it still maintains good transient-state characteristics.

## 4. Conclusions/Outlook

An asymmetric open D-dot voltage sensor is designed on the basis of the symmetric self-integral D-dot voltage sensor. It is more convenient and practical. The Ansoft Maxwell simulation demonstrates that the designed open D-dot voltage sensor causes small electric field distortion, but still could maintain satisfying insulating strength. According to the experimental test, this sensor has the potential for great accuracy and dynamic range. This research provides a new design idea of D-dot voltage sensor for the network voltage measurement. Nevertheless, the phase angle difference of the symmetric self-integral D-dot voltage sensor is discovered tob e very big, which will be further studied in the future.
